# Biosensors for Circular RNA Profiling in Biological Matrices

**DOI:** 10.1021/acs.analchem.6c01382

**Published:** 2026-07-13

**Authors:** Alessandra Glovi, Panagiota M. Kalligosfyri, Antonella Miglione, Gabriella Iula, Sima Singh, Michelino De Laurentiis, Stefano Cinti

**Affiliations:** † Scuola Superiore Meridionale, 9307 University of Naples Federico II, 80131 Naples, Italy; ‡ Department of Pharmacy, University of Naples Federico II, 80131 Naples, Italy; § Department of Public Health, University of Naples Federico II, 80131 Naples, Italy; ∥ Department of Breast and Thoracic Oncology, Istituto Nazionale Tumori IRCCS “Fondazione G. Pascale”, 80131 Naples, Italy; ⊥ Sbarro Institute for Cancer Research and Molecular Medicine, Center for Biotechnology, College of Science and Technology, Temple University, Philadelphia, Pennsylvania 19122, United States

## Abstract

Early detection of many diseases remains difficult because they often develop silently over the course of years. Circular RNAs are now at the forefront as biomarker candidates with a covalently closed structure, making them highly stable with exonuclease resistance and long half-lives. With their disease-specific expression pattern and their presence in biofluids, they offer easier and noninvasive monitoring of molecular signatures. Conventional detection techniques require centralized laboratories, sophisticated instrumentation, and specialized personnel, which restrict their widespread clinical adoption and limit their applicability in point-of-care diagnostic settings, but recent advances in biosensor technologies enable rapid, sensitive, and highly specific circRNA detection in biological matrices without complex equipment. Integration with nanomaterials, enzymatic amplification, and microfluidic or portable devices further enhances the specificity, signal strength, and clinical applicability. This Tutorial critically evaluates these emerging biosensing strategies, discusses current challenges, and provides practical guidelines for selecting circRNA biomarkers and corresponding detection methods. By bridging circRNA biology with advanced biosensor design, this work aims to accelerate translational research and guide the development of next-generation diagnostics for early disease detection, supporting a shift from reactive treatment to proactive health care.

## Graphical abstract

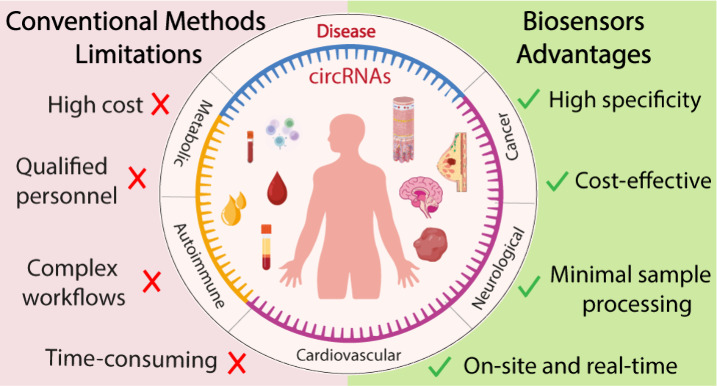

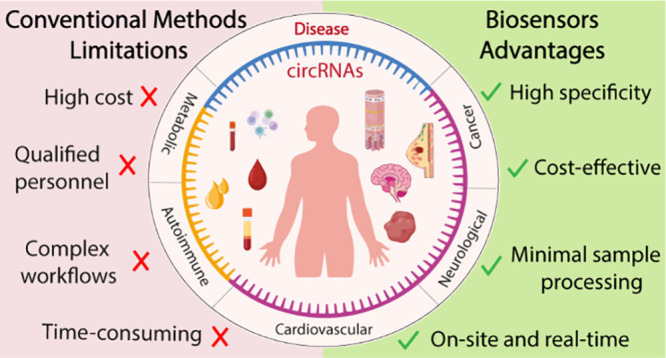

## Clinical Background and Emerging Role of CircRNAs

The landscape of molecular diagnostics is at a genuine inflection point. The development of disease pathobiology like cancer, neurodegeneration, cardiovascular, and autoimmune disorders often spans years before clinical symptoms emerge or imaging abnormalities become detectable. The development of amyloid and tau pathologies in Alzheimer’s disease (AD) begins before patients display any clinical symptoms, thereby narrowing the window for effective intervention once cognitive decline becomes apparent.^1,2^ The development of high-sensitivity cardiac troponin levels above normal ranges together with early autoimmune biomarkers shows that the disease has started to progress before patients show any signs of the disease.^3–5^ This increasing recognition of pre-symptomatic disease states has intensified the search for early biomarkers and driven the emergence of liquid biopsy approaches for early cancer detection and disease monitoring.^6^

Within this context, circRNAs emerge as diagnostic molecules, showing potential as biomarkers and demonstrating unique analytical and biological characteristics. These covalently closed single-stranded RNA molecules which form through non-canonical back-splicing of precursor mRNAs lack free 5′ and 3′ termini rendering them resistant to exonucleolytic degradation.^7^ Classic actinomycin D chase experiments revealed that circRNAs are much more stable than their linear mRNA counterparts, with half-lives often exceeding 48 h compared to less than 20 h for mRNAs.^8^ Importantly, circRNAs retain this stability throughout all stages of blood and clinical sample processing, from initial collection to final analysis.^9,10^ This exceptional stability, together with tissue-specific expression patterns and evolutionary conservation, positions circRNAs as highly robust and reliable indicators of pathology. In biological fluids, circRNAs can be detected both as free circulating molecules, where their covalently closed structure contributes to intrinsic resistance against degradation, and as cargo within extracellular vesicles, which may provide an additional layer of protection during circulation and sample processing (Figure 1). Consequently, depending on the biological matrix and analytical workflow, different sample preparation and enrichment or isolation strategies may be required prior to detection.^11^ circRNAs are known to actively participate in disease pathogenesis through multiple mechanisms including sequestering microRNAs as competitive endogenous RNAs, scaffolding protein complexes, controlling transcription and splicing and even encoding proteins through cap-independent translation pathways.^12^ Their functional versatility underlies their importance across a wide range of diseases (Figure 1).

**Figure 1:**
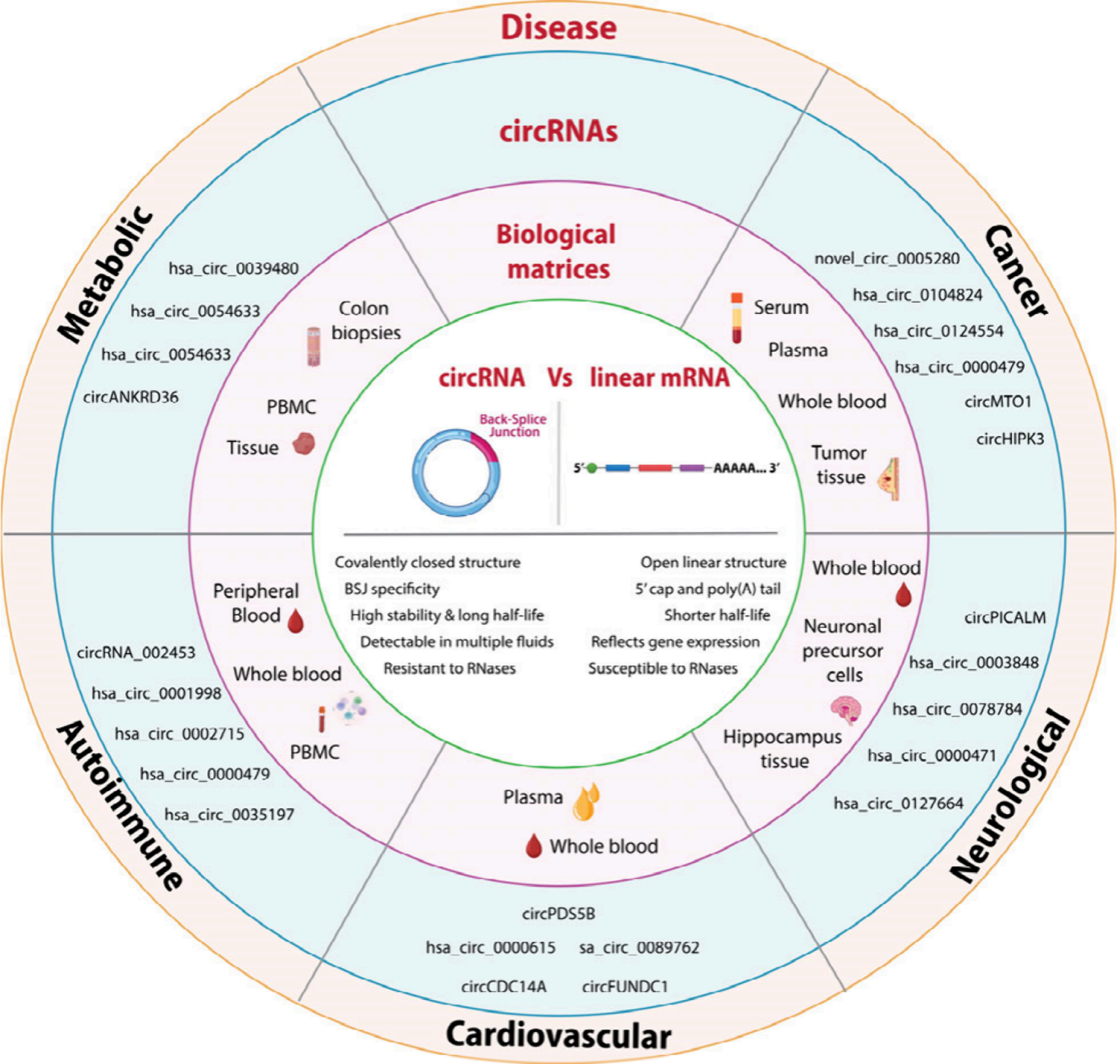
Schematic overview of disease-associated circRNAs and, biological matrices of detection, and key structural features relevant to biomarker applications. The outer ring classifies circRNAs according to major disease categories (metabolic, cancer, neurological, cardiovascular, and autoimmune disorders). The intermediate ring lists representative circRNAs reported in association with each disease group. The inner ring highlights the biological matrices in which these circRNAs have been identified, including tissues, blood-derived samples, and disease-relevant specimens such as tumor tissue or brain tissue. The central panel provides a simplified comparison between circRNAs and their linear mRNA counterparts, emphasizing differences in molecular structure, stability, RNase resistance, half-life, and biomarker-related properties.

Importantly, increasing evidence demonstrates that circRNAs have a strong prognostic value across multiple diseases. In cancer, circulating circRNAs are associated with patient outcomes by correlating with overall and disease-free survival, recurrence rates, metastasis, and treatment response, thereby enabling risk stratification of patients into low- and high-risk groups.^13^ In acute myeloid leukemia, dysregulated circRNA expression is linked to survival outcomes and clinicopathological features such as mutation status and disease aggressiveness.^14^ Similarly, in cardiovascular and neurodegenerative diseases, specific circulating circRNA signatures reflect disease severity and progression dynamics, allowing longitudinal monitoring of the disease course. Mechanistically, their prognostic relevance arises from their stability in circulation, disease-stage-specific expression patterns, and close association with key pathogenic pathways driving disease progression.^15^

In the nervous system they are highly abundant and dynamically regulated, serving as both disease biomarkers and regulators in neurodegenerative disease systems such as AD and Parkinson’s disease.^16,17^ In cardiovascular disease, they coordinate cardiomyocyte apoptosis and tissue remodelling, with specific circulating circRNA signatures indicate prognostic potential in cardiomyopathy risk mitigation and post-myocardial infarction risk stratification.^18,19^ circRNAs’ expression profile is significantly altered in autoimmune pathologies, where they actively regulate immune activation cascades and show strong diagnostic potential.^20^ Furthermore, in infectious diseases, such as COVID-19, host and viral circRNAs play immunomodulatory roles, as well as in metabolic diseases^21^ highlighting their dual utility as diagnostic biomarkers and potential therapeutic targets (Table 1).^22^

**Table 1:** Clinical Relevance of circRNA Biomarkers across Major Disease Classes and Biofluids

Disease class	Biofluid	Clinical relevance
Cancer	Blood (plasma/serum)	Early diagnosis, disease stratification, prognosis prediction, and treatment monitoring
Cardiovascular diseases	Blood (whole blood/plasma)	Risk stratification and outcome prediction (e.g., post-infarction and stroke prognosis)
Neurological diseases	Blood (plasma/serum) and brain-derived samples	Early detection and disease progression monitoring (emerging performance)
Autoimmune diseases	Blood (plasma/PBMC-derived fractions)^a^	Disease activity assessment and immune stratification
Metabolic diseases	Blood (serum/plasma)	Early metabolic dysfunction detection and disease progression monitoring
Inflammatory diseases	Blood (plasma)	Disease discrimination and severity-associated biomarker potential

a **PBMC:** peripheral blood mononuclear cells.

Although circRNAs hold great promise as biomarkers, their clinical translation is currently limited by critical analytical challenges. Detection methods such as RNA-seq and RT-qPCR are technically complex and resource-intensive, and circRNA concentrations often approach or exceed instrument sensitivity limits, making reliable quantification dependent on advanced amplification strategies.^23,24^ These analytical limitations can be overcome by integrating specific molecular recognition elements with advanced signal transduction platforms in biosensor systems. Modern biosensors achieve attomolar sensitivity and can complete analyses in an hour or less, even with complex biological samples, all performance features that position them as transformative tools for circRNA-based diagnostics.^25,26^

This Tutorial goes beyond a descriptive overview of circRNA detection and quantification methods^7^ by delivering a comprehensive biosensor-centered analysis of emerging technologies and architectures for circRNA profiling in complex biological matrices, critically examining their molecular recognition and signal transduction mechanisms, strengths, limitations, integration strategies, and translational potential for next-generation point-of-care (POC) diagnostics. Special attention is given to translational requirements, including analytical validation, integration into clinical workflows, and adherence to regulatory standards, which are essential steps for diagnostic implementation. By examining how these technological advances align with unmet clinical needs, we identify strategic priorities for the rational selection of circRNA biomarkers and corresponding sensing strategies, positioning biosensor platforms as key tools for precision medicine applications. In this way, this Tutorial serves as a practical framework for biomarker prioritization and analytical system design to facilitate clinical translation.

## Biosensor-Based Strategies to Address Current Limitations in CircRNA Detection

### Rationale for Biosensor-Based CircRNA Detection

The growing recognition of circRNAs as biologically informative biomarkers has intensified the demand for analytical platforms that can detect low-abundance targets with high specificity in complex biological matrices. Although circRNAs exhibit stability due to their covalently closed structure, their typically low expression levels and structural similarity to linear transcripts impose significant constraints on conventional detection techniques, particularly in liquid biopsy settings and longitudinal clinical monitoring.^11^ Amplification- and sequencing-based approaches remain powerful but often require multistep workflows, extensive sample processing, and specialized instrumentation, limiting their practicality for rapid or decentralized analysis.

Biosensor-based strategies have emerged as a promising solution by enabling direct molecular recognition of circRNAs coupled to real-time signal transduction, thereby reducing the assay complexity and analysis time. Several recent studies emphasize that biosensors can exploit the back-splice junction (BSJ) as a unique structural signature to achieve selective circRNA recognition, even in unamplified or minimally processed samples.^25,26^ This capability is particularly relevant for applications involving circulating circRNAs, where sensitivity, speed, and robustness are critical for reliable detection in plasma or other biofluids.^27^ Importantly, biosensors offer additional advantages beyond analytical sensitivity, including compatibility with miniaturized formats, potential for multiplexing, and adaptability to portable or POC platforms (Figure 2). These features position biosensor technologies as a complementary analytical layer that can bridge the gap between high-performance laboratory assays and clinically deployable circRNA detection tools, addressing the key limitations of existing methodologies while preserving molecular specificity.

**Figure 2:**
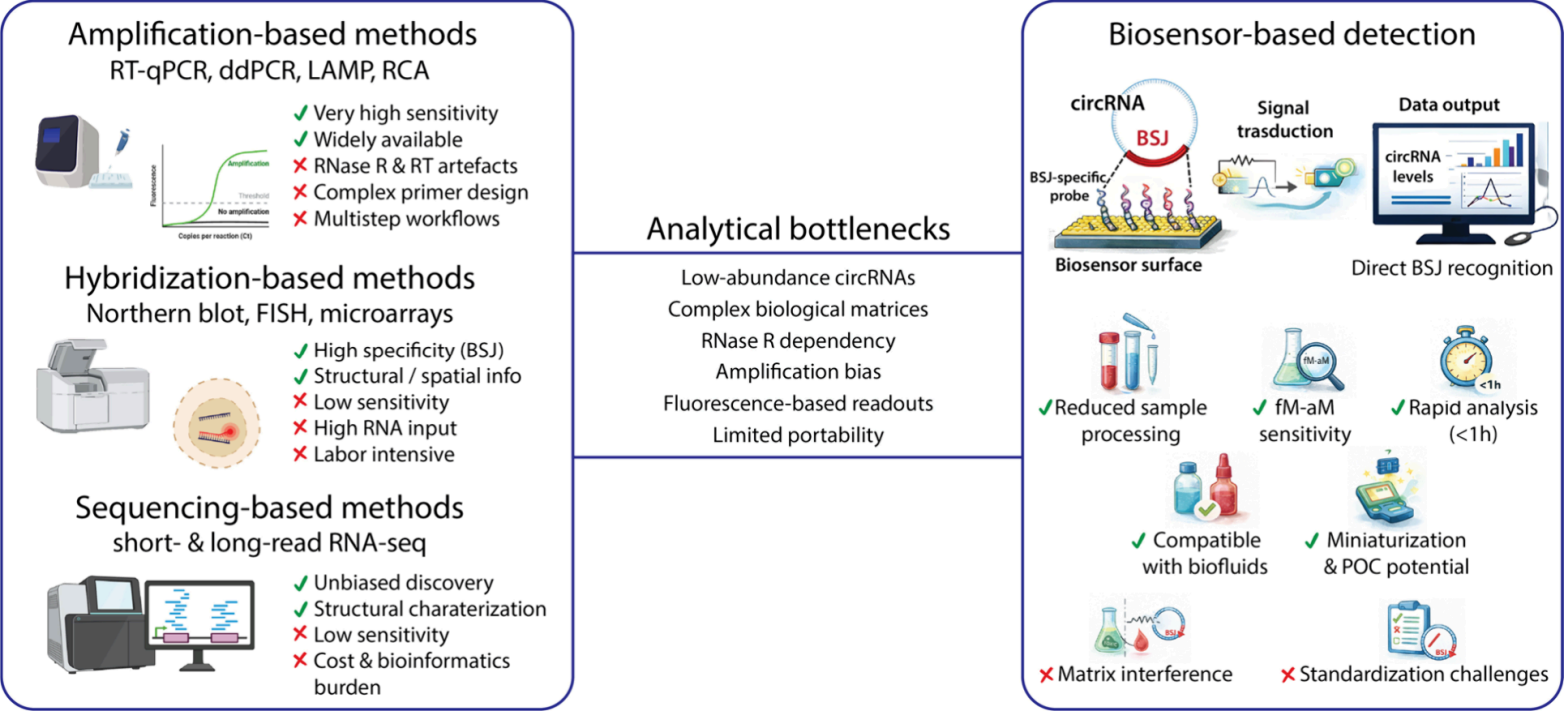
Overview of conventional and biosensor-based strategies for circRNA detection, highlighting analytical advantages, limitations, and key bottlenecks, as well as the potential of BSJ-targeted biosensors for sensitive and rapid analysis.

## Biosensing Strategies for CircRNA Detection

### Electrochemical Biosensors

Electrochemical biosensors are widely used for detecting nucleic acid and protein biomarkers in complex matrices such as serum, plasma, and whole blood. By avoiding optical instrumentation, they enable real-time and sometimes label-free detection with portable, cost-effective devices.^28,29^ Planar and screen-printed electrodes support multiplexing and direct analysis of minimally processed samples, while integrated signal- and target-amplification strategies enhance sensitivity without compromising selectivity.^30,31^

This flexibility makes electrochemical transduction well suited for circRNA detection, where low abundance and similarity to linear RNAs challenge conventional assays. CircRNA binding at functionalized electrodes generates amperometric, voltammetric, or impedimetric signals, with differential pulse and square-wave voltammetry commonly applied in BSJ-targeting and clustered regularly interspaced short palindromic repeats (CRISPR)-based formats.^32^

#### Hybridization-Based Approaches for Electrochemical Detection

In signal-off configurations, circRNA binding, typically mediated by BSJ-specific DNA or peptide nucleic acid (PNA) probes, leads to a decrease in the electrochemical signal because of steric hindrance, probe displacement, or disruption of redox-active labels at the electrode surface. A representative example of a signal-off, label-free electrochemical biosensor for circRNA detection was reported by Zhang et al.,^33^ who developed an electrochemical point-of-care testing (POCT) device for the detection of circ-CDYL in hepatocellular carcinoma (HCC). The platform employs a carbon-fiber microelectrode functionalized with gold nanoflowers, whose hierarchical three-dimensional nanostructure provides a significant increase of the electroactive surface area, thereby improving probe loading density and enhancing interfacial electron transfer kinetics. Thiolated PNA probes were immobilized through Au–S bonds to selectively bind the BSJ. Upon target binding, this label-free approach generates a measurable decrease in electrochemical current through the action of an added redox mediator. Target binding induces a measurable decrease in the electrochemical current. This enabled quantitative detection over a wide concentration range from 10 fM to 1 μM, with a limit of detection (LOD) of 3.29 fM. Thanks to the high affinity and specificity of the PNA probes, the sensor demonstrated single-base mismatch discrimination and was successfully validated for early-stage HCC diagnosis in human serum samples.

Signal-on electrochemical strategies increase the current upon circRNA binding. To detect low-abundance targets in serum, blood, or urine, nanomaterials such as gold nanoparticles (AuNPs) and metal–organic frameworks (MOFs) enhance probe density, charge transfer, and signal amplification while remaining stable and easily functionalized.

For example, Wu et al.^34^ developed a signal-on electrochemical platform using a dual-probe hybridization strategy and AuNPs-assisted redox signal amplification. A thiolated DNA capture probe (P1) was immobilized on the electrode, while a biotinylated probe (P2) hybridized to an adjacent target sequence, forming a sandwich-like recognition of the circRNA. Target binding promoted the attachment of ferrocene-capped AuNP/streptavidin conjugates, amplifying the voltammetric signal. The AuNP/streptavidin conjugates contributed to signal enhancement through their high surface-to-volume ratio and favorable electron-transfer properties, increasing the local concentration of redox-active ferrocene labels at the electrode interface. This system quantified circRNAs in breast cancer patients’ serum with low femtomolar sensitivity within 1–2 h, demonstrating the feasibility of nanoparticle-assisted signal-on detection in complex samples. The assay time was mainly determined by sequential hybridization and nanoparticle-incubation steps required for efficient signal amplification. Although this duration is longer than that of some recently developed rapid CRISPR-based electrochemical assays, it remains compatible with sensitive analysis in complex biological compares favorably with many established gold-standard molecular diagnostic approaches.^16^ Expanding on this approach, Liang et al.^35^ integrated MOFs into electrochemical biosensors for circ-HER2 detection. Circ-HER2-initiated rolling circle amplification (RCA) triggered self-assembly of PCN-222­(Fe) MOFs pre-loaded with electroactive molecules, achieving sensitive quantification from serum (0.1 fM LOD) and discriminating HER2-positive breast cancer from TNBC. The porous MOF architecture provided a high internal surface area for loading large quantities of methylene blue (MB) reporters, while the Fe-based nodes facilitated redox activity and promoted efficient charge transfer at the electrode interface. These advances illustrate the versatility of nanomaterial- and MOF-assisted signal-on architectures for sensitive circRNA detection. Another approach combined AuNPs, MOFs, and target amplification to further enhance sensitivity. A triple amplification strategy based on an Fe-MOF@AuNP-modified electrode achieved a detection limit as low as 0.046 fM.^36^ In this system, Fe-based MOFs decorated with AuNPs provided a conductive, high-surface-area interface, while RCA enabled target-induced signal amplification. Subsequent hybridization and catalytic steps, combined with electrochemiluminescence (ECL) detection using ruthenium-labeled probes immobilized via Au–S interactions, enabled ultra-sensitive identification of bladder cancer-associated circRNA-0137439 directly from urine samples, highlighting the potential of this strategy for non-invasive early diagnosis.

#### CRISPR-Based Approaches for Electrochemical Detection

CRISPR/Cas-based electrochemical biosensors enable highly sensitive, amplification-free circRNA detection with rapid, low-detection limit voltammetric readouts, suitable for POC applications and real-time monitoring. Among CRISPR-powered electrochemical biosensors reported to date, Cheng et al.^37^ developed a Cas13a-based platform for the detection of bladder cancer-associated circRNA in urine samples. In this sensing platform, DNA tetrahedron (TDN) nanostructures were used to precisely organize MB-labeled RNA reporter probes at the electrode interface. The rigid and well-defined three-dimensional geometry of the TDNs improved probe orientation and accessibility while minimizing steric hindrance and enhancing interfacial electron-transfer efficiency compared with conventional surface immobilization strategies. Upon hybridization of the target circRNA, Cas13a induced collateral trans-cleavage of the surface-confined RNA reporters, leading to a decrease in the electrochemical signal. Notably, the assay provides results within 10 min, confirming its strong potential for rapid on-site analysis and POC bladder cancer diagnosis (Figure 3A).

**Figure 3:**
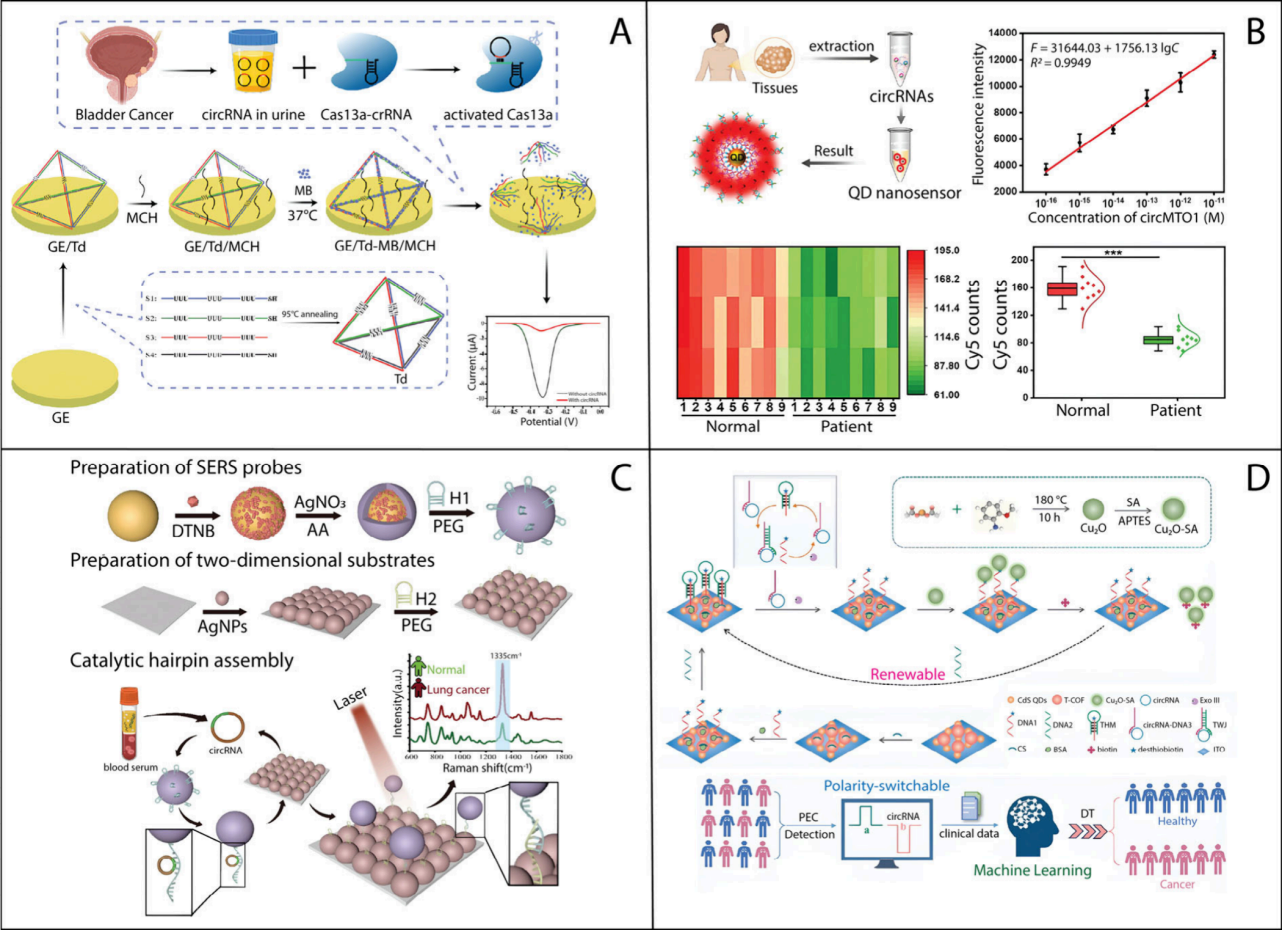
Representative biosensor-based strategies for circRNA detection. A) CRISPR-Cas13a-assisted electrochemical biosensor for urinary circRNA detection in bladder cancer. Reprinted with permission from ref 37. Copyright 2025 Elsevier. B) Fluorescence-based quantum dot nanosensor for circRNA quantification in tissue samples. Reprinted with permission from ref 43. Copyright 2024 Elsevier. C) SERS-based biosensing platform combining catalytic hairpin assembly and nanostructured substrates for serum circRNA detection. Reprinted with permission from ref 23. Copyright 2024 American Chemical Society. D) Integrated electrochemical biosensing system with renewable sensing interface and machine-learning-assisted circRNA analysis for clinical classification. Reprinted with permission from ref 54. Copyright 2024 Elsevier.

Overall, among electrochemical biosensing strategies, signal-on architectures appear to be particularly promising for circRNA detection because they provide stronger signal amplification, lower background interference, and improved sensitivity for low-abundance targets in complex biological matrices. In contrast, signal-off approaches, although simpler to design, rely on signal suppression from a high baseline and are constrained by limited dynamic signal changes, making them more susceptible to background fluctuations and false-positive responses caused by unintended probe loss. Signal amplification approaches such as RCA and CRISPR/Cas, together with nanomaterials including AuNPs and MOFs, substantially improve analytical performance. However, multistep workflows and prolonged biomarker isolation or enrichment procedures remain major barriers to clinical translation.

### Optical Biosensors

#### Fluorescence-Based Biosensors

Fluorescence-based approaches enhance circRNA detection by monitoring intensity changes, supporting solution-phase, spatial, and multiplexed assays through versatile probe designs using fluorophores, quenchers, and fluorescent nanomaterials. In circRNA applications, fluorescence transduction commonly relies on fluorophore–quencher systems (e.g., molecular beacons, hairpin probes) or Förster resonance energy transfer (FRET) mechanisms, where BSJ-mediated hybridization restores fluorescence. To achieve sufficient sensitivity in complex biological matrices, most fluorescent circRNA biosensors integrate cyclic signal amplification (CSA) strategies, which convert single binding events into multiple fluorescence outputs under isothermal, low-instrumentation conditions.^38^ Widely adopted amplification schemes include RCA, hybridization chain reaction (HCR), strand displacement reactions (SDR), and enzyme-assisted amplification cascades.

#### Hybridization/Amplification Approaches for Optical Detection

Building upon CSA concepts, multi-layered nucleic acid amplification architectures have been developed that integrate isothermal amplification with DNA self-assembly. Dong et al.^39^ reported a signal-on fluorescence platform in which RT-RCA is coupled with a netlike HCR cascade, producing extended DNA nanostructures that collectively activate FRET-based hairpin probes. This hierarchical amplification strategy achieved sub-picomolar sensitivity while maintaining high selectivity for the BSJ sequence. It demonstrates that combining coordinated amplification with DNA nanostructure assembly can substantially enhance fluorescence signals without compromising assay specificity. In the context of enzymatic target amplfication, Qu et al.^40^ reported a homogeneous fluorescence assay using FAM/BHQ1-labeled molecular beacons combined with T7 exonuclease-assisted cyclic enzymatic amplification. In this approach circRNA recognition induces repeated beacon cleavage, releasing fluorophores and generating amplified fluorescence signals. This enzyme-driven signal cycling achieved a LOD of 1 pM for circRNA in buffer and was successfully applied to semiquantitative analysis of circRNA in tumor cell lysates, demonstrating the effectiveness of molecular beacon-based fluorescence amplification for low-abundance targets.

Fluorescent nanostructures have also been exploited to improve probe organization, signal-to-noise ratio, and robustness in biologically relevant matrices. Beyond conventional intensity-based measurements, fluorescence lifetime analysis represents a particularly advantageous implementation of FRET for circRNA biosensing. Since the donor fluorophore lifetime is shortened upon energy transfer to an acceptor, circRNA recognition events can be transduced through lifetime variations rather than absolute fluorescence intensity changes. Lifetime-based approaches are less dependent on fluorophore concentration and more resistant to photobleaching, optical scattering, and background autofluorescence, potentially improving analytical robustness in complex biological matrices and single-cell analysis settings.^41,42^ The relevance of fluorescence lifetime analysis in circRNA biosensing was experimentally demonstrated by Liu et al., who reported a quantum dot-based nanosensor achieving attomolar detection limits (10^–18^ M) with a wide linear range from attomolar to picomolar levels. The platform successfully differentiated circRNA expression in breast cancer tissues versus adjacent normal counterparts and enabled single-cell circRNA analysis, with results comparable to qRT-PCR analysis. In addition, measurable shortening of the 605QD donor lifetime from 35.98 to 17.71 ns upon circMTO1 recognition experimentally confirmed FRET occurrence through fluorescence lifetime analysis. However, key limitations include multiple temperature-dependent enzymatic steps (42 and 37 °C), the need for specialized single-molecule imaging systems, and validation primarily in extracted RNA and spiked serum rather than minimally processed clinical samples. Although such ultra-low detection limits highlight the analytical capability of advanced circRNA biosensors, their practical clinical necessity remains context-dependent, and existing practical constraints may limit translation to routine POC use despite their high sensitivity.^43^ (Figure 3B) In this category, DNA tetrahedral nanoprobes (DTNPs) provide well-defined spatial arrangements of fluorophores and quenchers, enhanced probe accessibility, and improved resistance to nonspecific interactions. In particular, Xie et al.^44^ leveraged the intrinsic microRNA sponge property of circRNAs using a DTNP functionalized with Cy3-labeled miRNA recognition sequences and BHQ2 quenchers, achieving fluorescence signal recovery in a serum-mimicking environment containing 10% fetal bovine serum. Although the reported sensitivity remained in the picomolar range, this study highlighted the feasibility of circRNA fluorescence sensing in protein-rich matrices and underscored the influence of matrix complexity on assay performance.

Integrated fluorescence-based sensors for circRNAs are still largely lacking, but He et al.^25^ addressed this gap by developing the first microfluidic chip for circRNA detection. The platform combines tetrahedral DNA nanostructures (TDNs) as probe scaffolds with target-initiated HCR amplification. BSJ-targeting probes were organized on the PDMS microchannel surfaces via TDNs, improving accessibility, reducing nonspecific adsorption, and enhancing stability. circRNA binding triggered an isothermal HCR, generating amplified fluorescence signals within the microfluidic channels. The device achieved a 0–200 aM linear range, 19 aM LOD, required only 100 μL sample, remained stable for one month, and accurately quantified circRNAs from cancer cell RNA, closely matching RT-qPCR measurements.

#### CRISPR-Cas-Based Approaches for Optical Detection

As previously discussed, CRISPR/Cas-based systems offer enhanced selectivity and specificity compared with conventional nucleic acid detection. This stems from a programmable mechanism in which target binding to a CRISPR RNA (crRNA) activates the Cas effector, triggering collateral nuclease cleavage and signal amplification. In circRNA detection, this recognition is typically coupled to optical readouts, often combined with isothermal or enzyme-free amplification to improve sensitivity without PCR. For example, Song et al.^45^ designed a Cas13a-based exponential amplification system for circRNA detection. In this approach, BSJ recognition triggers Cas13a activation, which initiates successive stem–loop-mediated amplification and LAMP reactions. This strategy enables sensitive detection of circRNAs in biological samples, achieving a 1 fM LOD while maintaining strict discrimination against linear RNA isoforms.

To further streamline assay workflows and reduce operational complexity, Ke et al.^46^ introduced an integrated CRISPR–RCA sensing strategy, in which RT-RCA generates repeated BSJ-containing units that efficiently activate Cas12a trans-cleavage, yielding fluorescence readouts with attomolar sensitivity (∼300 aM) and compatibility with cell-derived RNA samples.

Beyond conventional fluorophore–quencher reporters, Liu et al.^47^ exploited Cas13a trans-cleavage to release a trigger sequence from a rU-containing substrate probe upon recognition of the target circHIPK3. The trigger initiates cyclic primer-exchange reactions (PER) with gated hairpins, generating numerous single-stranded G-quadruplexes. Thioflavin T selectively intercalates into these structures, producing strongly amplified fluorescence. By autonomously converting target recognition into optically active G-quadruplexes, the system achieves ultrasensitive circRNA detection down to 0.83 aM, minimizes nonspecific background, and enables profiling in single cells and clinical tissue specimens, demonstrating robustness in complex biological environments.

Finally, Liu et al.^48^ developed a mirror-synchronized asymmetric CRISPR-Cas12a nanoswitch for circRNA detection using two palindromic hairpin probes (PHP1 and PHP2) that bind the BSJ and initiate bidirectional cyclic amplification. The resulting activators trigger an asymmetric CRISPR-Cas12a cascade, and a Cy5/BHQ2-labeled probe converts activity into fluorescence. This approach enables ultrasensitive, one-pot circRNA detection (1.07 aM LOD) with single-mismatch BSJ discrimination.

#### Surface-Enhanced Raman Spectroscopy (SERS) Biosensors

Raman spectroscopy offers molecular fingerprints but is limited by weak scattering. SERS uses nanostructured gold or silver to amplify signals via plasmon resonance, enabling ultrasensitive, multiplexed circRNA detection.^49^ SERS performance depends on the plasmonic substrate and probe placement in enhanced electromagnetic regions. For circRNA detection, BSJ-targeting oligonucleotide probes are often paired with enzyme-free amplification to boost low-abundance signals. The circRNA SERS biosensors reported to date predominantly employ label-based detection strategies, in which Raman-active reporter molecules are incorporated directly into the plasmonic nanostructure (typically within the gap between metallic shells in core–shell or core–satellite architectures) rather than relying on intrinsic Raman scattering of the target molecule itself. This design ensures a stable and reproducible Raman signal confined within electromagnetic hot spots, decoupling signal generation from direct target adsorption on the plasmonic surface. Regarding substrate design, several key factors govern SERS performance in biological matrices. First, hot-spot reproducibility is crucial: core–satellite architectures with controlled interparticle spacing produce more uniform enhancement than random aggregates, improving assay reproducibility. Second, biofouling in serum or whole blood, due to nonspecific protein adsorption on gold or silver surfaces, can reduce signal intensity and increase background noise; thus, surface passivation (e.g., PEG or compact DNA layers) is essential. Third, internal Raman standards embedded within the nanostructure enable ratiometric normalization and compensate for substrate and matrix variability. Finally, magnetic-assisted target enrichment enhances sensitivity and selectivity by concentrating circRNA–probe complexes prior to SERS readout.^50,51^ For example, Xu et al.^23^ created a SERS-based optical nanobiosensor using 2D-SERS substrates and catalytic hairpin assembly (CHA) to detect circSATB2 linked to lung cancer in human serum. In this platform, a hairpin chain H2 was immobilized on the 2D substrate via sulfhydryl groups to form SERS nanoprobes. The circRNA target opened the hairpin H1 on the SERS probe, exposing its 5′ end, which then competitively unfastened H2 and formed H1-H2 double strands. This sandwich-type assembly immobilized the Raman-active-labeled SERS probe on the substrate, amplifying the readout signal (Figure 3C). The platform achieved ultrasensitive detection with an LOD of 0.766 fM and could distinguish lung cancer patients from healthy controls, including specific stage and subtype profiles, highlighting its potential as a noninvasive early diagnostic and prognostic tool.

A similar principle has been applied to magnetic-assisted core–satellite architectures, where controlled plasmonic hot spots and magnetic target enrichment enhance SERS signal reliability in serum-based circRNA analysis. Using a CHA-assisted magnetic SERS platform, Au@MBA@Ag nanoprobes and Fe_3_O_4_@Ag magnetic nanoparticles formed hot spots, while internal-standard self-calibration enabled one-pot detection of lung cancer-related circRNAs. This design achieved an ultra-low LOD of 5.5 aM and reliably distinguished early-stage (IA/IB, IA1–IA3) lung cancer in human serum.^52^

Beyond extracellular detection, SERS biosensors have also been adapted for intracellular circRNA analysis. Xu et al.^53^ developed a dual-signal SERS nanoprobe using a 4-MBN internal standard and ROX reporter for quantitative imaging of circSATB2 in living lung cancer cells. This system achieved a 0.043 pM LOD and was validated against RT-PCR. Incorporating internal standards and narrow-band Raman reporters enables signal normalization, multiplexing, and robust detection in heterogeneous cellular environments, demonstrating SERS’s potential for high-resolution spatial profiling of circRNAs at the single-cell level.

#### Colorimetric Biosensors

Colorimetric biosensors enable circRNA detection through visible color changes that can be read by the naked eye or simple devices such as portable photometers or smartphone-based imaging systems. Their operational simplicity, low cost, and minimal instrumentation requirements make them attractive for decentralized and field-deployable nucleic acid analyses.^32^

Recent advances in nanotechnology have expanded colorimetric circRNA biosensors, particularly through AuNPs, whose plasmonic, size-dependent optical properties enable visible color changes. The size-dependent localized surface plasmon resonance of AuNPs produces a visible red-to-blue colorimetric shift upon nanoparticle aggregation, enabling naked-eye detection while enhancing analytical sensitivity through controlled interparticle spacing and DNA-directed assembly. CircRNA recognition modulates AuNP aggregation or dispersion via hybridization, while structured probe architectures improve assembly control, sensitivity, and background suppression. For instance, oriented aggregation strategies based on Y-shaped DNA duplexes enable more precise regulation of interparticle distances, leading to substantial improvements in detection sensitivity compared with conventional salt-induced aggregation schemes.^55^ Colorimetric circRNA biosensors have also incorporated catalytic signal amplification to enhance response intensity and robustness. As an illustrative example, Li et al.^56^ developed a colorimetric nanobiosensor for hepatocellular carcinoma-associated circRNA cSMARCA5 that integrates BSJ-specific molecular recognition with magnetic bead-assisted purification of complex samples. In this platform, magnetic nanoparticles (MNPs) functionalized with BSJ-specific hairpin probes were employed to selectively capture and isolate the circRNA target from complex biological matrices (i.e., whole blood and tissue matrices) prior to detection, effectively reducing background interference from non-target biomolecules and improving assay sensitivity. Subsequent circRNA binding initiates a CHA cascade that generates multiple G-quadruplex–hemin DNAzyme units per target molecule, producing an amplified colorimetric signal. The biosensor achieved a LOD of 1.93 fM across a dynamic range from 10 fM to 100 nM and enabled accurate circRNA analysis in tissue and blood samples using a simple colorimetric readout. This work illustrates how coupling molecular amplification with sample pretreatment can improve the applicability of colorimetric biosensors in biologically complex matrices.

### Field-Effect Transistor (FET) Biosensors

Biosensors based on field-effect transistor (FET) architectures enable label-free nucleic acid detection by transducing target-induced changes in surface charge into measurable variations in the electrical conductance of a semiconducting channel. Owing to their high surface-to-volume ratio and direct electrical readout, FET platforms, particularly nanowire and nanoribbon geometries, have been widely explored for DNA and RNA sensing.^57^ However, their application to circRNA detection remains limited. To date, the only reported example is by Ivanov et al.^58^ who reported a nanoribbon-based silicon-on-insulator (SOI) field-effect transistor biosensor for the label-free detection of the glioma-associated circRNA circNFIX. In this platform, DNA oligonucleotide probes complementary to the circNFIX sequence were covalently immobilized on the surface of SOI nanoribbons, and target recognition was transduced into real-time changes in the drain–source current of the FET channel. Owing to the high surface-to-volume ratio of the nanoribbon architecture, the biosensor achieved an experimentally determined LOD up to the sub-femtomolar level. The response was recorded within 15 min without the use of labels or enzymatic amplification. The biosensor was validated with circRNA from human plasma, detecting elevated circNFIX in glioma patients versus healthy and disease controls, confirming FET-based detection in relevant biological samples.

### Photoelectrochemical Biosensors

Beyond the widely used electrochemical and optical readouts, circRNA biosensing has been successfully extended to photoelectrochemical (PEC) transduction to enhance analytical robustness in complex biological matrices. In this context, Yuan et al.^24^ integrated CRISPR/Cas13a-mediated circRNA recognition with Cu nanocluster reporters and a Z-scheme T-COF/Ag_2_S photoactive substrate. Upon specific binding of the circRNA target to the crRNA, Cas13a activation induces collateral cleavage of Cu nanocluster-modified probes, modulating charge-transfer processes at the PEC interface and generating a measurable photocurrent response. This architecture enabled highly sensitive target detection directly in whole blood, achieving a LOD in the low-attomolar range while maintaining excellent specificity under biologically relevant conditions. Extending this concept, a machine learning-assisted, polarity-switchable PEC biosensor uses Exo III-driven recycling and Cu_2_O nanospheres to switch between anodic and cathodic photocurrents. In this system, the Cu_2_O nanospheres act as a p-type semiconductor component whose surface density, regulated by the amplification cascade, modulates the dominant charge-transfer pathway at the photoactive interface, thereby enabling the polarity-switchable photocurrent response. The platform reached a 7.6 aM LOD for circSATB2 in whole blood and delivering 100% accuracy, sensitivity, and specificity for distinguishing lung cancer patients from healthy individuals^54^ (Figure 3D).

Among optical circRNA detection platforms, integrated fluorescence and CRISPR/Cas-assisted strategies currently demonstrate the strongest potential, owing to their high programmability, excellent BSJ specificity, and ultra-high analytical sensitivity. Fluorescence systems combined with amplification approaches such as RCA, HCR, and CRISPR-mediated cascades consistently achieve femtomolar- to attomolar-level detection. In parallel, SERS-based platforms provide additional advantages for multiplexed analysis and single-cell profiling through plasmon-enhanced signal enhancement. Despite these advances, many optical platforms still depend on complex multistep amplification schemes, specialized instrumentation, and time-intensive circRNA isolation or enrichment processes. Moving forward, greater emphasis should be placed on developing streamlined and integrated systems capable of maintaining sensitivity while reducing the operational burden and facilitating broader clinical implementation.

### Current Landscape of circRNA Biosensors

Among the reported circRNA biosensing strategies (summarized in Table 2), optical sensors, particularly fluorescence-based platforms, account for more than 60% of the total studies. Fluorescent biosensors offer flexible probe design, modular labeling, and strong compatibility with amplification-driven sensitivity enhancement, supporting the low-abundance circRNA detection. However, integrated fluorescence-based circRNA sensors remain largely lacking, and their application in complex clinical matrices is limited by background autofluorescence, probe degradation, optical scattering, and long reaction times. Consequently, most assays have been validated in cell lysates or serum-mimicking systems rather than in minimally processed biofluids. Electrochemical biosensors represent a scalable, ultrasensitive, and potentially portable approach, enabling the real-time, label-free detection of circRNAs in complex biological matrices. Most reported designs rely on immobilized probes, often supported by nanostructures, i.e., AuNPs and MOFs, to enhance probe density, charge transfer, and signal amplification. Although probe immobilization enables sensitive and label-free transduction, it introduces practical constraints, including limited long-term probe stability, batch-to-batch variability, and reduced target accessibility in complex biological matrices, where nonspecific adsorption and biofouling can compromise signal integrity and assay reproducibility.^59^

**Table 2:** Overview of Representative Biosensor Platforms Reported for circRNA Detection, Summarizing the Transduction Principles, Target CircRNAs and Associated Diseases, Biological Matrices, Amplification Strategies, Biosensor Modification, Detection Times, Temperature, Relative Standard Deviation (RSD%), Limits of Detection (LOD) and Recovery (%)^a^

Assay principle/Detection	Target circRNA/Disease	Biological matrix	Amplification strategy	Biosensor modification	Detection time/Temperature	LOD/RSD (%)	Recovery (%)	Ref
**Electrochemical Biosensors**								
*Hybridization-Based Approaches*								
Signal-off PNA-based hybridization assay on SPE; DPV readout	Circ-CDYL/HCC	Human serum	AuNFs -enabled signal enhancement	CFME modified with AuNFs and thiolated PNA probes	7 h/RT	3.29 fM (Buffer)/2.1 (*n* = 3)	N/R	^33^

Signal-on sandwich-type hybridization strategy with Fc-capped gold nanoparticle/streptavidin conjugates; CV readout	CircAHNAK/TNBC	PCR-amplified products from human whole blood	Fc-capped AuNP/streptavidin binding to biotinylated DNA	Gold electrode modified with thiolated DNA probe (P1); biotinylated probe (P2); Fc-capped gold nanoparticle/streptavidin conjugates	6.5 h/RT	0.22 pM (Buffer)/<5 (*n* = 3)	N/R	^34^

Signal-on DNA-based hybridization assay enabling RCA initiation; SWV readout	Circ-HER2/Breast cancer	Human serum	RCA-mediated cascade combined with MB@MOF-TDN nanoprobes	MB@MOF-TDN nanoprobes; Gold electrode	7 h/up to 37 °C	0.1 fM (Buffer) /N/R	N/R	^35^

Signal-on DNA-based hybridization assay; ECL readout	Has-circRNA-0137439/Bladder cancer	Human urine	Multiple signal amplification strategies, including RCA, quadruple amplification, and cyclic amplification	Fe-MOF@AuNPs-modified electrodes	5 h/up to 30 °C	0.046 fM (0.1 M PBS pH 7.4 + 0.1 M TPrA)/<7 (*n* = 3)	93.3–118.81	^36^

Signal-on DNA probe-based hybridization assay on SPME; DPV readout	Circ-CDYL/HCC	Human serum	MBs-assisted target enrichment; AuNPs-enabled signal enhancement	AuNPs-SPME; Biotinylated DNA probes on MBs; MB redox intercalator	<3 h/*RT*	1.0 pM (Buffer) /< 9 (*n* = 3)	115.1–119.7	^64^

*CRISPR-Based Approaches*								
Signal-off CRISPR/Cas13a-based assay using collateral cleavage of Td-MB complex on a gold electrode; DPV readout	Bladder-cancer-associated circRNA/bladder cancer	Human urine	CRISPR/Cas13a collateral cleavage-driven signal amplification	Td/MCH-modified gold electrode; MB redox intercalator	<10 min/RT	0.089 fM (Buffer)/7.4 (*n* = 3)	N/R	^37^

Signal-on CRISPR/Cas13a-based assay with MB-mediated signal transduction; SWV readout	cZNF292/AMI	Whole blood	PER-mediated cascade coupled with CRISPR/Cas12a collateral trans-cleavage; AuNPs-enabled signal enhancement	CB[7]@GE (PDDA/AuNPs/CB[7]) electrode; MB-DNA signal probe	1 h/RT	2.13 fM (Buffer)/4.09 (*n* = 3)	93.55–107.0	^65^

**Optical Biosensors**								
*Fluorescent Biosensors – Hybridization-Based Approaches*								
Signal on TDN- based hybridization on a microfluidic chip/Fluorescence intensity readout	Circ_006127/Gastric cancer	Total RNA extracted from cancer cells (AGS cells)	Hybridization chain reaction (HCR) signal amplification	Tetrahedral DNA nanostructure (TDN)-modified PDMS microfluidic chip	1 h/RT	19 aM (Buffer)/N/R	N/R	^25^

Signal-on detection via FRET hairpin probe activation; Fluorescence intensity readout	CircTEX2/HCC	Extracted cellular RNA from cell lysates	Dual nucleic acid amplification via RT-RCA coupled with netlike HCR	N/A	5 h/up to 85 °C	0.1 pM (Buffer)/3.73– 17.82 (*n* = 3)	N/R	^39^

FAM and BHQ1-labeled MBs; Fluorescence intensity readout	CircBART2.2/EBV-associated cancers	Extracted cellular RNA from cell lysates	T7 exonuclease-assisted cycling enzymatic amplification	N/A	3 h/37 °C	1 pM (Buffer) /3.7 (*n* = 3)	N/R	^40^

QD-Cy5 FRET modulation; Fluorescence intensity readout	CircMTO1/BC	Spiked human serum (1% and 5%)	TWJ-mediated cascade amplification (SDA + PG-RCA)	DNA three-way junction-guided functionalization of quantum dots	3 h/up to 95 °C	7.12 aM (Buffer)/3.22 (*n* = 3)	98.1–104.3	^43^

Signal-on DTNP based on Cy3/BHQ2 quenching and recovery; Fluorescence intensity readout	CircMTO1/NSCLC	Spiked 10% FBS	miRNA sponge amplification property of circRNAs	DTNP functionalized with Cy3-labeled miRNA-760 recognition sequences and BHQ2 quenchers	1 h 15 min/37 °C	84.54 pM (10% FBS)/N/R	N/R	^44^

DNAzyme-mediated cleavage-induced signal recovery	CircMTO1/HCC	Whole blood	Triple amplification cascade (CHA + PER + DNAzyme)	N/A	2 h/37 °C	0.265 fM (Buffer)/N/R	91–100	^66^

*Fluorescent Biosensors – CRISPR-Based Approaches*								
CRISPR/Cas13a-induced exponential amplification; Fluorescence intensity readout via SYBR Green intercalation	ciRS-7 (CDR1as)/cancer-associated circRNA	Total RNA extracted from HT29 cell lysates	CRISPR/Cas13a-mediated catalytic primer release triggering LAMP-based exponential amplification	N/A	1 h/up to 65 °C	1 fM (Buffer)/N/R	102.6–104.5	^45^

FAM fluorescence recovery via ssDNA reporter cleavage; Fluorescence intensity readout	CircRNA1445/HCC	Extracted cellular RNA from human hepatocyte (LO2) and hepatocellular carcinoma (HepG2) cell lysates	RT-RCA generation of BSJ repeat units coupled with CRISPR/Cas12a collateral cleavage amplification	N/A	2 h/up to 85 °C	300 aM (Buffer)/N/R	N/R	^46^

Thioflavin T light-up fluorescence via G-quadruplex formation; Fluorescence intensity readout	CircHIPK3/breast cancer	Total RNA extracted from cancer cell lines and clinical breast tissue samples	CRISPR/Cas13a-mediated trans-cleavage triggering PER cascade amplification	N/A	40 min/37 °C	0.83 aM (Buffer)/N/R	N/R	^47^

Mirror-synchronized asymmetric CRISPR nanoswitch realizing Cy5 photon/Camera sensor detecting and quantifying single photon events	CircMTO1/breast cancer	Extracted RNA from human breast cancer cells	Dual-palindromic hairpin-mediated exponential enrichment coupled with asymmetric CRISPR/Cas12a trans-cleavage cycling	N/A	25 min/37 °C	1.07 aM (Buffer)/2.64 (*n* = 10)	95.9–103.0	^48^

*SERS Biosensors*								
Signal-on DNA-based hybridization; Ratiometric Raman readout	CircSATB2/Lung cancer	Human serum	CHA-mediated enzyme-free cyclic signal amplification	Core–shell Au@DTNB@Ag SERS nanoprobe and Ag NP 2D substrate functionalized with DNA hairpins	2.5 h/37 °C	0.766 fM (Buffer)/4.71–5.39	N/R	^23^

Signal-on DNA-based hybridization; Ratiometric Raman readout	CircVAPA/Lung cancer	Human serum	CHA-mediated enzyme-free cyclic amplification	Magnetic core–satellite SERS platform based on Fe_3_O_4_@Ag and Au@Ag nanostructure	2 h/37 °C	5.5 aM (Buffer)/<7	92.1–101.3	^52^

Signal-off DNA-based competitive hybridization; Ratiometric Raman readout	CircSATB2 (BSJ)/Lung cancer	Extracted cellular RNA from cell lysates	N/A	Dual-signal Au@4MBN@Au SERS nanoprobes with embedded internal Raman standard	24 h/37 °C	0.043 pM (Buffer)/3.96 (*n* = 10)	95.6–101.6	^53^

*Colorimetric Biosensors*								
Formation of G-Quadruplex-Hemin DNAzyme DNAzyme-catalyzing TMB oxidation; Absorbance readout	cSMARCA5/HCC	Whole blood	CHA cascade coupled with G-quadruplex–hemin DNAzyme amplification	MNPs functionalized with BSJ-specific hairpin probes	3.5 h/up to 80 °C	1.93 fM (Buffer)/N/R	N/R	^56^

**FET Biosensors**								
Label-free DNA probe-modified nanoribbon chip; Electronic readout	CircNFIX/glioma	Extracted plasma RNA	N/A	SOI nanoribbon FET chip functionalized with complementary DNA probes	<10 min/RT	0.011 fM (Buffer)/N/R	N/R	^58^

**PEC Biosensors**								
Photocurrent quenching; PEC readout	CircSATB2/Lung cancer	Extracted whole-blood RNA	CRISPR/Cas13a trans-cleavage–triggered HCR and dsDNA-templated in situ Cu nanocluster generation	Z-scheme T-COF/Ag_2_S photoactive composite on ITO electrode/Cu nanoclusters as photocurrent quencher	4 h/37 °C	0.5 fM (Buffer)/N/R	98.2 −103.1	^24^

Polarity-switchable; PEC readout	CircSATB2/Lung cancer	Extracted whole-blood RNA	TWJ–Exonuclease III-assisted cyclic amplification	CdS/T-COF photoactive layer, DNA probes and Cu_2_O nanospheres	2 h/37 °C	7.6 aM (Buffer)/N/R	N/R	^54^

a **Abbreviations: aM**: attomolar; **Ag**_**2**_ **S**: silver sulfide; **AMI**: acute myocardial infarction; **AuNPs**: gold nanoparticles; **BSJ**: back-splice junction; **CHA**: catalytic hairpin assembly; **CFME**: carbon fiber microelectrode; **COF**: covalent organic framework; **CRISPR**: clustered regularly interspaced short palindromic repeats; **Cu NCs**: copper nanoclusters; **Cu**_**2**_ **O**: cuprous oxide; **DPV**: differential pulse voltammetry; **ECL**: electrochemiluminescence; **Exo III**: exonuclease III; **FBS**: fetal bovine serum; **FET**: field-effect transistor; **fM**: femtomolar; **HCC**: hepatocellular carcinoma; **HCR**: hybridization chain reaction; **ITO**: indium tin oxide; **LAMP**: loop-mediated isothermal amplification; **LOD**: limit of detection; **MB**: methylene blue; **ML**: machine learning; **MNPs**: magnetic nanoparticles; **miRNA**: microRNA; **N/A**: not applicable; **N/R:** not reported; **PEC**: photoelectrochemical; **PER**: primer exchange reaction; **PNA**: peptide nucleic acid; **PoC/POCT**: point-of-care/point-of-care testing; **qPCR**: quantitative polymerase chain reaction; **RCA**: rolling circle amplification; **RT-RCA**: reverse transcription–rolling circle amplification; **SARS-CoV-2**: severe acute respiratory syndrome coronavirus 2; **SDA**: strand displacement amplification; **SERS**: surface-enhanced Raman scattering; **SOI**: silicon-on-insulator; **SPR**: surface plasmon resonance; **SWV**: square wave voltammetry; **TDF**: tetrahedral DNA framework; **ThT**: thioflavin T; **TMB**: 3,3′,5,5′-tetramethylbenzidine; **TPrA**: tripropylamine; **TWJ**: three-way junction; **UV–vis**: ultraviolet–visible spectroscopy; **Z-scheme**: Z-scheme heterojunction charge-transfer architecture.

Importantly, a major limitation across nearly all current platforms is that they predominantly operate in a single-target detection mode rather than in true circRNA profiling systems. In clinically relevant settings, disease classification is rarely driven by individual circRNAs but instead depends on multivariate expression signatures composed of several dysregulated circRNAs. Therefore, the transition from single-analyte detection to multiplexed circRNA profiling represents a critical unmet need in this field. At the same time, only one CRISPR-assisted electrochemical assay has been reported, highlighting a significant opportunity for integrating programmable recognition for improved specificity and multiplexing. Notably, recovery and RSD values were more consistently reported in electrochemical and SERS-based biosensors, reflecting the comparatively more advanced analytical validation achieved with these platforms. By contrast, many emerging CRISPR-based and optical proof-of-concept systems primarily emphasized signal amplification strategies and ultra-low detection limits, while more comprehensive evaluation of assay robustness and clinical validation parameters remains comparatively limited.

From a systems-level perspective, circRNA diagnostics should therefore evolve toward integrated profiling platforms that combine multiplex detection, standardized quantification, and multivariate interpretation of circRNA expression patterns. Such an approach would enable the reconstruction of disease-specific circRNA “fingerprints” rather than isolated biomarker readouts.

In this context, the most important advance in circRNA biosensing is the shift toward fully integrated hybrid platforms that combine CRISPR-mediated programmable recognition with isothermal nucleic acid amplification and nanomaterial-assisted signal transduction, enabling attomolar detection directly in complex clinical biofluids and significantly advancing the field toward clinically relevant, POC diagnostic applications.

Despite this progress, current limitations of all reported biosensors include the lack of multiplexed panels, limited integration with upstream sample processing, and the need for robust portable devices. Lessons from liquid biopsy diagnostics of other biomarkers, such as microRNAs or antibodies, suggest that microfluidic integration, passive fluid transport, pre-concentration, and reagent storage could substantially improve circRNA detection workflows.^60–62^ Other platforms, such as colorimetric, FET, and PEC biosensors, remain at early stages of development, with only a few of them reported to date. The limited progress in colorimetric sensors is understandable given LOD constraints, yet it is surprising that they are lagging despite their low cost, highlighting the inherent complexity of circRNA detection. SERS offers unparalleled sensitivity and molecular fingerprinting capabilities, but reproducibility, signal variability, and data processing challenges limit clinical translation.^63^ FET biosensors have demonstrated sub-femtomolar detection but face obstacles in high-ionic-strength biological fluids, including Debye screening and nonspecific adsorption. PEC biosensors, often combined with CRISPR, have achieved attomolar sensitivity in whole blood, yet wider adoption has been limited.

CircRNA profiling across biological matrixes represents a key interface between disease biology and biosensor design. As summarized in Table 2, most clinically relevant circRNA signatures are currently derived from blood-based matrices, including plasma, serum, and whole blood, reflecting the minimally invasive nature and translational accessibility of the liquid biopsy approaches. In addition, alternative matrices such as urine in urogenital malignancies and brain-derived or RNA-enriched fractions in neurological disorders are gaining attention, although they remain underexplored in biosensing applications. Peripheral blood mononuclear cell (PBMC)-derived fractions further provide an intermediate compartment that captures immune-related transcriptional alterations, which is particularly relevant in autoimmune and inflammatory diseases.

From an analytical perspective, circRNA profiling extends beyond target identification to encompass the coordinated consideration of biological matrix selection, pre-analytical processing, and sensing architecture design. These three parameters jointly govern the assay performance, reproducibility, and clinical relevance. Notably, most reported biosensors are still validated under simplified conditions such as serum or spiked buffer systems, underscoring the need for systematic evaluation under clinically realistic biofluid complexity and disease-specific molecular backgrounds.

Accordingly, next-generation circRNA biosensing is shifting toward integrated analytical frameworks that combine multiplexed circRNA panel detection, cross-matrix validation across diverse biofluids, and longitudinal or time-resolved measurements of biomarker dynamics. This transition enables a move from single-analyte quantification to holistic analytical platforms that integrate selectivity, matrix robustness, and temporal resolution within a unified sensing strategy.

## Future Perspectives and Conclusions

Future development of circRNA biosensors should focus on the seamless integration of amplification strategies, CRISPR-based recognition, and nanomaterial-enhanced signal transduction into miniaturized, portable, and user-friendly devices. A major gap in current platforms is the lack of multiplexing capabilities, which is critical for liquid biopsy applications where circRNA panels, rather than single targets, serve as combinatorial biomarker signatures. Implementing multiplexed detection would allow simultaneous profiling of multiple circRNAs, increasing diagnostic accuracy and enabling comprehensive disease characterization.^37^

To achieve real-world applicability, upstream sample handling must be seamlessly incorporated, including strategies such as pre-concentration, filtration, reagent storage, and passive fluid transport, especially in paper-based and microfluidic devices, which are essential to maintain assay sensitivity for low-abundance circRNAs in complex biological matrices such as plasma, serum, urine, or cell lysates.^67^ Automated workflows combined with chemometric and AI-assisted design-of-experiment optimization strategies may improve probe designs, amplification parameters, and signal readouts, reducing variability, improving reproducibility, and enabling high-throughput analysis.^68,69^ Importantly, AI-assisted circRNA profiling systems can enable the conversion of multiplex biosensor outputs into predictive diagnostic models, facilitating patient stratification, disease staging, and treatment response prediction.^70^

Portability remains another critical limitation, as many currently reported platforms still rely on laboratory-based instrumentation, limiting practical POC implementation. Miniaturized thermal cyclers, integrated microfluidic chips, or paper-based fluidics can facilitate rapid, low-volume sample handling and detection in decentralized settings. Furthermore, isothermal enzymatic amplification and enzyme-free strategies should be prioritized to allow rapid, sensitive detection without lengthy multistep protocols, which is a necessity given the inherently low abundance of many circRNAs.

Finally, successful clinical translation will require strong interdisciplinary collaboration among engineers, chemists, biologists, and clinicians as many circRNAs remain emerging biomarkers with limited large-scale clinical validation. Future guidelines should emphasize multiplexed panels, optimized probe design, seamless sample processing, portable devices, amplification-friendly workflows, and clinically informed assay development to bridge the gap between laboratory innovation and actionable POC diagnostics.

Overall, circRNA biosensors represent a rapidly evolving field with significant potential for next-generation liquid biopsy diagnostics. Although remarkable analytical sensitivity has already been achieved across multiple sensing modalities, broader clinical implementation will depend on improvements in multiplexing capability, assay standardization, integrated sample handling, and real-world validation in complex biological matrixes. Continued advances in biosensor engineering, molecular amplification, and portable analytical technologies are expected to accelerate the translation of circRNA-based diagnostics toward practical precision medicine and POC applications.
